# Clinical Results of Distal Radius Intraarticular Comminuted Fractures in the Elderly: A Retrospective Comparative Analysis of Different Fixation Methods

**DOI:** 10.7759/cureus.28077

**Published:** 2022-08-16

**Authors:** Ozcan Kaya, Deniz Gulabi, Halil Buyukdogan, Ali Can Baris, Bulent Kilic, Mustafa Caliskan

**Affiliations:** 1 Orthopaedics and Traumatology, Sağlık Bilimleri Üniversitesi (SBU) Istanbul Kanuni Sultan Suleyman Training and Research Hospital, Istanbul, TUR; 2 Orthopaedics and Traumatology, Istanbul Haydarpasa Training and Research Hospital, Istanbul, TUR

**Keywords:** wrist function, clinic outcomes, external fixation, volar plate fixation, distal radius fracture, intraarticular comminuted fractures

## Abstract

Introduction

Distal radius intraarticular fractures in the elderly population are likely to cause impaired clinical outcomes. Intraarticular fracture treatment in the literature is a debatable issue with mixed results. Here, we aimed to present a tertiary trauma center experience with plate fixation and K wire-assisted external fixator in elderlies over 60 years old.

Material and methods

Patients who were diagnosed with an unstable intraarticular distal radius fracture and received surgical treatment with plate fixation or K wire-assisted external fixator between 2016 January and 2020 January were included in the study. Patients were evaluated retrospectively in terms of radiologic stability criteria and clinical outcomes.

Results

There were 27 patients; 14 in the volar plate group (group 1) and 13 (group 2) in the external fixator group. The mean age was 64.2 (60-72) in group 1 and 67.7 (60-76) in group 2. The mean follow-up time was 31.6 (12-63) in group 1 and 28.8 (12-59) in group 2. The mean quick disabilities of the arm, shoulder, and hand (Q-DASH) score was 25.7 (5-75) in group 1 and 24.4 (10-87) in group 2. The mean patient-reported wrist evaluation (PRWE) was 27.1 (6-87) in group 1 and 31.4 (10-87) in group 2. There was no statistical difference between groups in terms of clinical scores, hospital stay, follow-up, and complications. (p>0.05).

Conclusion

Although open reduction and plate fixation and K wire-assisted external fixator are viable options for providing radiologic union, unsatisfactory clinical outcomes were maintained independently of the fixation method in elderly patients.

## Introduction

Distal radial fractures are commonly diagnosed as upper extremity fractures in emergency department visits. In the US, approximately 44% of upper extremity fractures are distal radius fractures [1-4]. In younger patients, distal radius fractures are usually caused by high-energy trauma, and in the elderly, distal radius fractures are caused by low to moderate-energy trauma with a background of osteopenia or osteoporosis. High-energy trauma seems to be mostly related to comminuted or intraarticular fractures, whereas low to moderate-energy trauma results in metaphyseal fractures [3-4].

High-energy-related distal radius fractures are mostly seen in men as compared to women. Women suffer distal radius fractures especially in the postmenopausal period due to a reduction of bone mineral density. The overall age-adjusted incidence of distal radius fractures is greater in women than men. An increase in life expectancy in the elderly population results in a higher incidence of distal radius fractures and the need for surgical or nonsurgical management increased over time [5-7].

The treatment of distal radius fractures depends on various factors. Fracture morphology and stability, age, hand dominance, and patient comorbidities are well-known factors that affect the treatment choice. Fractures that are not intraarticular, noncomminuted, and stable mostly benefit from conservative treatment [6-7]. Open reduction and stable fixation is the preferred treatment choice for intraarticular displaced fractures for favorable long-term clinical outcomes. The communition of fracture and stability signs after initial reduction provides guidance to definitive treatment methods. In some cases, it may be necessary to choose different treatment methods other than open reduction & internal fixation (ORIF) in the treatment of these fractures due to variable patient factors [8-15].

 In the literature, there are mixed results of different treatment methods for comminuted intraarticular fractures. The success of treatment is based on anatomic reduction and stable fixation of the fracture. In the present study, we aimed to compare retrospectively clinical results of two fixation methods for complex intraarticular comminuted distal radius fractures.

## Materials and methods

Upon receiving institutional board approval, the patient medical archiving system search was performed with International Classification of Diseases (ICD) code S52.5 between 2016 and 2020. We reviewed the medical records of 52 patients who underwent surgery for distal radius fractures. Inclusion criteria for patients were defined as: 1. patients over 60 years old, 2. isolated wrist fracture, 3. treated with one of the above-mentioned surgical fixation methods (volar locking plate = group 1; external fixation = group 2). Inclusion criteria for fracture morphology are defined as intra-articular displaced comminuted fractures. Patients with a history of additional ipsilateral or contralateral upper extremity surgery due to the same traumatic event, with a follow-up smaller than one year, were excluded.

**Figure 1:**
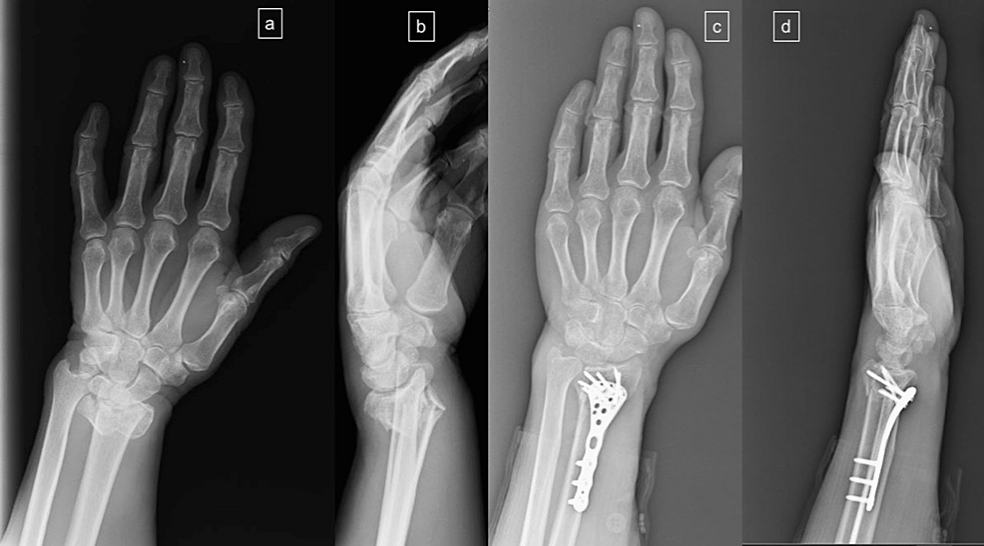
A 68-year-old male with an intraarticular comminuted distal radial fracture treated with a volar locking plate a: Preoperative anteroposterior X-ray of the wrist; b: Preoperative lateral X-ray of the wrist intraarticular and volar metaphyseal fragments; c: Open reduction and fixation with a distal anatomic locking plate d: Lateral X-ray of the wrist

Surgical procedures for group 1 were performed via a volar modified Henry approach between the radial artery and the flexor carpi radialis tendon. The approach is deepened between the flexor pollicis longus and the radial artery. The pronator quadratus muscle was incised in an L shape, reduction was performed under fluoroscopy, and fixation was performed with a locking anatomical distal radius plate (Figure 1).

**Figure 2:**
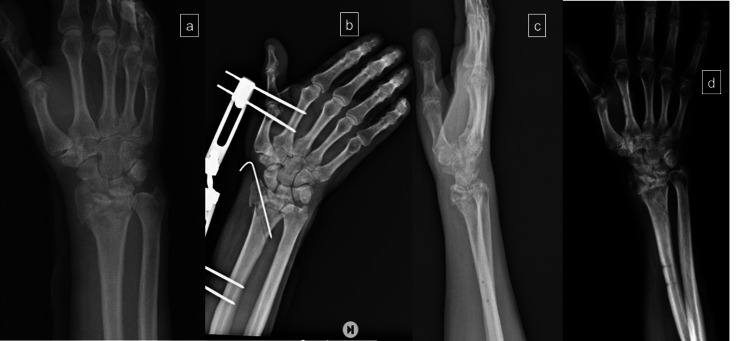
A 76-year-old female underwent K wire-assisted external fixator a: Preoperative anteroposterior X-ray of the wrist; b: Closed reduction and fixation with K wire-assisted wrist external fixator; c: lateral X-ray of the wrist after removal of the fixator and K wires; d: AP X-ray of the wrist after the removal of the K wires and external fixator AP: anteroposterior

In group 2, the surgical procedure was performed according to this sequence: closed reduction under fluoroscopy and K wire fixation were performed and followed by a wrist joint spanning unilateral external fixator, which was kept in a semi-flexed and ulnar position; 4.0 mm pins were inserted in the second metacarpal shaft (pin insertion points were proximal to the transition of the metacarpal head into the shaft) and the radius shaft (pin insertion points were proximal to the muscle bellies of the abductor pollicis longus (APL) and extensor pollicis brevis (EPB)) (Figure 2).

All patients who were in group 1 used three-week volar cast support with a wrist intrinsic plus position and fingers left free at the metacarpophalangeal joints. After removal of the supportive cast, wrist joint exercises were initiated. In group 2, joint rehabilitation could not be initiated until the removal of the external fixator and K wires. Wrist and finger range of motion exercises were initiated according to patient pain response and fixation type.

Surgery-related complications, presence of union, failure of fixation, limitation of wrist joint range of motion, and at the last follow-up, the quick disabilities of the arm, shoulder, and hand (Q-DASH) and patient-reported wrist evaluation (PRWE) scores were recorded for clinical and radiological outcome measures.

Statistical analysis was performed with SPSS 25.0 (IBM Corp., Armonk, NY). Descriptive statistics were performed with minimum maximum and mean values for continuous variables (eg; age, Q-DASH). Proportions were given for categorical variables (sex, complications). The Kruskal-Wallis test was utilized within a 95% confidence interval and p-value <0.05 to test for statistical significance. The Mann-Whitney U test was used to investigate whether there is a statistical significance between groups.

## Results

The number of patients according to inclusion criteria was 27. In group 1, there were 14 patients (8F; 6M) with a mean age of 64.21 ± 3.06 (between 60 and 72) and in group 2, there were 13 patients (8F, 5M) with a mean age of 67.69 ± 5.67 (between 60 and 76). Almost two-thirds of fractures occurred after a fall from a standing height.

The mean hospital stay time was 2.5 ± 1.78 (between 1 and 7 days) in group 1 and 4.23 ± 3.44 (between 1 and 10 days) in group 2. The mean follow-up time was 31.6 ± 35.7 (12-63) months in group 1 and 28.8 ± 12.7 (15-59) months in group 2.

There was no statistical difference between groups in terms of age, sex, follow-up time, and hospital stay (p>0.05).

Health-related quality of life (HRQOL) scores were mildly affected in both groups. The mean VAS score was 7.5 ± 1.45 (between 4 and 9) in group 1 and 7 ±1.41 (between 4 and 9) in group 2. Quick DASH evaluation in the last follow-up was 25.7 ± 22 (between 5 and 75) in group 1 and 24.42 ± 22.7 (between 5 and 75) in group 2. The mean PRWE score was 27.14 ± 25.2 (between 6 and 87) in group 1 and 31.4 ± 21.3 (between 10 and 87) in group 2. HRQOL scores seemed to be worse in group 2 but this finding was not statistically meaningful. (VASp = 0.181;Q-DASHp = 0.313; PRWEp = 0.346). Due to the small number of patients in the groups and with the aim of not excluding extreme cases, mean values were used.

None of the patients in both groups received a second surgery due to nonunion. In group 2, there were three (23%) patients with pin site infection treated with wound care and oral antibiotics. The loosening of Schanz pins was not recorded. Wrist joint arthritis developed in two (14%) patients in group 1 and 2 patients in group 2 (15%). The limitation of range of motion was recorded in two (14%) patients in group 1 and two (15%) patients in group 2 (Table 1).

**Table 1:** Demographics and comparative analysis of variables between groups Group 1: Volar plate group; Group 2: K wire-assisted external fixator group; DASH: disabilities of the arm, shoulder, and hand (0 = no disability; 100 = most severe disability); VAS: visual analog scale; PRWE: patient-rated wrist evaluation (0=disability; 100=worst functional score); ROM: range of motion; NA: not applicable

Variable	Group 1	Group 2	P value
Age, (years)			
Mean±SD	64.21±3.06	67.69±5.67	.242
Range	60-72	60-76	
Sex,#			
Male	6	5	1.000
Female	8	8	
Hospital stay (Days)			
Mean±SD	2.5±1.78	4.23±3.44	.804
Range	1-7	1-10	
Follow-up (months)			
Mean±SD	31.64±35.7	28.84±12.7	.275
Range	12-63	15-59	
Quick DASH			
Mean±SD	25.71±22	24.42±22.71	.313
Range	5% - 75%	5% - 75%	
VAS			
Mean±SD	7.5±1.45	7±1.41	.181
Range	4-9	4-9	
PRWE			
Mean±SD	27.14±25.2	31.46±21.34	.346
Range	6-87	10-87	
Complications:			
Nonunion	-	-	NA
Infection	-	3 (23%)	NA
Arthritis	2 (14%)	2 (15%)	NA
Limited ROM	2 (14%)	2 (15%)	NA

## Discussion

There are different treatment options for comminuted, intraarticular fractures of the distal radius. It’s mandatory to analyze patient characteristics, activity demands, fracture stability, and displacement for making a decision to choose the best approach [1,5-14]. To date, it’s seen that open reduction and fixation via a volar approach is the most common approach for these fractures. Here, we present our experience with intraarticular distal radius fractures among the elderly above 60 in a tertiary trauma center. In our cohort, although the union was achieved in all patients independent of fixation type, clinical satisfaction was slightly negatively affected in both groups.

Distal radius fractures with/without ulnar involvement are the most seen fractures of the upper extremities in the emergency department. Distal radius fractures (DRF) are usually caused by high-energy trauma in young adults, whereas, in the elderly, low to moderate-energy trauma causes distal radial fractures. High energy-related DRF in younger adults may involve greater articular involvement and comminution. Men are more prone to be affected by high-energy trauma-related DRF. Despite this, the overall incidence of DRF is greater in women than in men [1-5]. However, the incidence of comminuted intraarticular fractures increases with advancing age [1-2]. In our present study, despite sustaining only low-energy trauma, our patients' fractures were classified into subtype C under the Arbeitsgemeinschaft für Osteosynthesefragen (AO) classification. The distribution of cases based on sex, men and women, was equal.

Management of DRF in the elderly depends on various factors, and clinic results are debatable. In the literature, conservative treatment of DRF may also give satisfactory results independent of the remaining radiologic deformity with an acceptable range of motion and forearm rotations [6-7].

Moreover, some reports concluded that insufficient anatomic repositioning may not be correlated with a higher degree of disability while some authors indicated that clinical outcomes and radiologic findings are correlated. In contrast, in younger individuals, it’s a well-studied fact that inadequate reduction is strongly related to unsatisfied clinic results. It’s very difficult to figure out whether clinic results in the elderly may be attributed to normal age-related decreased function or radiologic appearance and diminished skeletal integrity due to age [3-4,7].

Surgical treatment of DRF has evolved with time. The aim of the surgery for DRF is to restore articular incongruity for improved clinic results [1,8-9]. In the elderly population, this effect still remains unclear. In long-term follow-up, studies reported that articular incongruity resulted in arthritis and mild to moderate functional limitation [3-4]. Surgical treatment options are external fixation, dorsal plate fixation, volar plating, bridge plating, and fragment-specific fixation. There is no advised exact surgery approach and fixation type for fractures, therefore surgeons should be familiar with all methods and choose the appropriate fixation method according to fracture type [1,5,8-9].

The introduction of the volar distal radius anatomic locking plate (VLP) resulted in a shift toward its use for daily surgical practice. Applying a VLP via the volar Henry approach requires minimal soft tissue dissection compared to dorsal plate fixation [10-11]. Complications such as tendon irritation and ruptures caused by plate prominence are very rarely seen [15]. However, the easy application and availability of most of the DRFs that are severely comminuted with dorsal shear fragments and involving the dorsal ulnar corner may not be suitable for VLP [5,8-9]. We use VLP for volar shear fractures, with radial shortening of more than 3 mm, a dorsal tilt greater than 10º, and more than a 2 mm articular step-off. In the present cohort, we used VLP with moderate dissatisfaction in the Q-DASH and PRWE scores and low to moderate complication rates. There was the development of arthritis in two (14%) patients and a limited range of motion. Union was achieved in all patients. The daily activities of all patients were not limited.

External fixation with/without the K wire combination aims to maintain fracture reduction with ligamentotaxis and control angulation and displacement in DRF [1,16-17]. In younger patients with a comminuted intraarticular fracture, it has been shown that external fixation provides improved radiographic and functional outcomes when compared with a plaster cast [17]. Besides these, in previous reports on elderly patients, especially in osteoporotic bones, external fixator use was found to be less effective to maintain radial length. Clinic and radiologic outcome results are mixed in the literature [1,10,12,14,17]. Kreder et al. reported that in the external fixator group, return to function and relief of pain were more rapid than in the open reduction surgery group [18]. In a randomized prospective trial comparing VLP and external fixator by Roh et al., no difference in grip strength, range of motion, and functional outcome were found between treatment groups [19]. In our department, we prefer to use an external fixator in highly comminuted intraarticular fractures and unstable polytrauma patients. Complications related to external fixator treatment are pin tract infection, small joint stiffness, and nerve injury. In our cohort, we recorded three (23%) patients with pin tract infections who were treated with oral antibiotics without removal of pins. Arthritis (15%) and limited range of motion (15%) were observed in two patients. Our complication rate was comparable with the literature. The daily activities of all patients were not limited due to these complications, and bone healing was achieved in all patients.

Our study has several limitations. The first notable limitation of our study was its retrospective nature and the limited number of patients. In the elderly, most DRFs are managed conservatively due to different reasons. Although there is an increasing trend in using VLP, external fixator application indications are very limited so the small number of the cohort can be explained by these concerns. DRFs in the elderly draw attention to osteoporosis but our patients were not evaluated according to bone mineral density. In the elderly, as concluded in previous studies, the evaluation of clinic outcomes is very difficult, and attribution of the disability to advanced age or fracture characteristics is still controversial.

## Conclusions

Although open reduction and plate fixation and K wire-assisted external fixation are viable options for providing radiologic union, unsatisfactory clinical outcomes were maintained independently of the fixation method in elderly patients. VLP seems to provide better function in wrist and radiologic parameters with lower complication rates at early follow-up but it's not significant in the present study due to the lower number of patients. At follow-ups of more than one year, both fixation methods have similar radiologic and clinic results.
